# Changes in Prescribing Symptomatic and Preventive Medications in the Last Year of Life in Older Nursing Home Residents

**DOI:** 10.3389/fphar.2017.00990

**Published:** 2018-01-23

**Authors:** Helene G. van der Meer, Katja Taxis, Lisa G. Pont

**Affiliations:** ^1^Unit of PharmacoTherapy, Epidemiology and Economics, Groningen Research Institute of Pharmacy, University of Groningen, Groningen, Netherlands; ^2^Discipline of Pharmacy, Graduate School of Health, University of Technology Sydney, Sydney, NSW, Australia

**Keywords:** end of life, aged-care, nursing homes, medication use, deprescribing, palliative care, drug utilization, preventive medicine

## Abstract

**Background:** At the end of life goals of care change from disease prevention to symptomatic control, however, little is known about the patterns of medication prescribing at this stage.

**Objectives:** To explore changes in prescribing of symptomatic and preventive medication in the last year of life in older nursing home residents.

**Methods:** A retrospective cohort study was conducted using pharmacy medication supply data of 553 residents from 16 nursing home facilities around Sydney, Australia. Residents received 24-h nursing care, were aged ≥ 65 years, died between June 2008 and June 2010 and were using at least one medication 1 year before death. Medications were classified as symptomatic, preventive, or other. A linear mixed model was used to compare changes in prescribing in the last year of life.

**Results:** 68.1% of residents were female, mean age was 88.0 (*SD*: 7.5) years and residents used a mean of 9.1 (*SD*: 4.1) medications 1 year before death. The mean number of symptomatic medications per resident increased from 4.6 medications 1 year before death to 5.1 medications at death [95% CI 4.4–4.7 to 5.9–5.2, *P* = 0.000], while preventive medication decreased from 2.0 to 1.4 medications [95% CI 1.9–2.1 to 1.3–1.5, *P* = 0.000]. Symptomatic medications were used longer in the last year of life, compared to preventive medications (336.3 days [95% CI 331.8–340.8] versus 310.9 days [95% CI 305.2–316.7], *P* = 0.000).

**Conclusion:** Use of medications for symptom relief increased throughout the last year of life, while medications for prevention of long-term complications decreased. But changes were slight and clinical relevance can be questioned.

## Introduction

At all stages across the life span, the decision to prescribe a medication should be based on weighing potential benefits and harms of the medication considering the individual’s treatment goals. Goals range from decreasing mortality and morbidity, prevention of future conditions or complications, or minimization of symptoms. Toward the end of life, in addition to considerations around potential medication related benefits and harms, treatment choice should also take life expectancy into consideration. As life expectancy decreases, the goals of care may change from decreasing mortality and morbidity, to symptom control (Holmes et al., 2006). Long-term residential aged care or nursing home residents are among the frailest of all older populations. They are generally medically complex, using a high number of medications, and this complexity together with age-related pharmacokinetic- and dynamic puts them at high risk of adverse outcomes related to medication (Taxis et al., 2016, 2017; Australian Institute of Health and Welfare, 2017).

Of all aged care residents, 91% die in the nursing home after an average stay of 168 weeks for women and 110 weeks for men, indicating that the majority of residents have limited life expectancy following nursing home admission (Australian Institute of Health and Welfare, 2017). Adjusting prescribing according to a decreasing life expectancy involves deprescribing, defined as the process of withdrawing inappropriate medications (Lee et al., 2013; Scott et al., 2015). Hence, in this population a decrease in preventive and an increase in the use of medications for symptom control and palliation could be expected (Maddison et al., 2011).

To date few studies exploring changes in the use of symptomatic and preventive medications have been conducted in older nursing home populations at the end of life. A recent systematic review found that use of preventive medications in patients with limited life expectancy was common (Poudel et al., 2017). Only few studies focused on deprescribing and there was no consensus on how to optimize medication use at the end of life. Of the 15 studies included, three were performed in a nursing home setting (Poudel et al., 2017). These studies included only a small study population (Blass et al., 2008) or had a cross-sectional study design (Onder et al., 2013; Heppenstall et al., 2016). In order to consider optimization of medication use at the end of life we need to understand the current patterns of use as life expectancy decreases. Therefore, the aim of this study was to explore changes in prescribing of symptomatic and preventive medications in the last year of life among older nursing home residents.

## Materials and Methods

### Study Design and Setting

A retrospective cohort study of 3876 nursing home residents living in 26 residential aged care (RAC) facilities in New South Wales, Australia between 1st June 2008 and 10th June 2010. The RAC facilities varied from low care to high care. High care facilities provided 24 h nursing care including medication administration. All residents received medical care from the general practitioner of their choice and were eligible to receive annual medication reviews by a pharmacist. Each facility has a contracted pharmacy for medication supply.

### Study Population

Recruitment was done at the facility level. All residents aged 65 years or older who died in one of the 26 RAC facilities between 2nd of June 2008 and 10th of June 2010 were included in the cohort. To allow medication changes in the year prior to death to be explored, only those residents who were taking at least one medication 1 year prior to death were included in the cohort. Residents who were discharged prior to death were excluded from the study, as medication use could not be ascertained once they left the facility.

### Data Source

Weekly pharmacy medication supply data, including all prescription, non-prescription and complementary medications, were used for the study. The dataset included generic name, dose, date of commencement, date of cessation and if the use was regular or *‘as needed.’* The dataset also included limited demographic data for each resident including age, sex, date of admission, date and reason for discharge and facility.

### Medication Classification

Medications were coded using the World Health Organization Anatomical Therapeutic Chemical (ATC) code (WHO Collaborating Centre for Drug Statistics Methodology, 2016a). Medications were classified into three categories: symptomatic, preventive, and other. All medications recommended for symptom control in the Australian national palliative care guidelines were considered as symptomatic medications (Palliative Care Expert Group, 2010; Australian Government Department of Health, 2015). Medications defined in the literature for primary or secondary prevention of all-cause mortality were defined as preventive medications (Hilmer et al., 2012). Preventive medications included antihypertensive medications (NPS Medicinewise, 2016), antithrombotic agents (WHO Collaborating Centre for Drug Statistics Methodology, 2016b), osteoporosis medication (NPS Medicinewise, 2017), and lipid modifying agents (WHO Collaborating Centre for Drug Statistics Methodology, 2016c). Medications that were not considered as either preventive or symptomatic were classified as other. Antibiotics, topical preparations, ophthalmological and otological medications were excluded due to the episodic nature of the use of these medications. Vaccines were also excluded as they were administered by the general practitioner and not supplied by the pharmacy. A list of included medications can be found in the Appendix.

### Outcomes

Three main outcome measures were determined. First, we compared the mean number of symptomatic, preventive, and other medications per resident at 1 year, 6 months, 1 month, and 1 week (8 days) before death and on the day of death. Second, we compared the type of symptomatic, preventive, and other medication used 1 year before death versus on the day of death. For this analysis we included all medications, grouped by ATC level 2, which were used by at least 10% of the population either 365 days before death or on the day of death. Third, we compared the duration of use of symptomatic, preventive, and other medications in the last year of life. We included all medications used 365 days before death, and calculated the days of treatment during the last year of life.

All medications used 7 or fewer days before death were considered to be taken on the day of death. This was done for two reasons. First, medication was supplied per week, therefore the last medication might have been supplied up to 7 days before death. Second, we assumed some inaccuracies in recording the date of death due to a delay in nursing home staff notifying pharmacy staff.

### Statistical Analysis

Medication changes were analyzed with a linear mixed model to account for clustering of medications within one resident. Our data did not allow clustering for general practitioners. Therefore we performed clustering on the level of facility, to account for possible intra-facility culture of medication prescribing. We included a random intercept and a random slope at the level of resident and facility in the analysis. Analyses were adjusted for age, gender, duration of admission and number of medications at 365 days before death, if the individual *P*-value in the univariate analysis was 0.25 or less (Bursac et al., 2008). The number of medication and days of treatment were reported as estimated marginal means with their 95% confidence intervals. The second outcome was analyzed using a McNemar test. We report on proportions and absolute numbers of residents. All analyses were conducted in IBM SPSS 24 on a significance level of 0.05.

### Ethics Statement

This study was approved by the Sydney South West Area Health Service Human Research Ethics Committee, the Concord Repatriation General Hospital (CH62/6/2010-49 HREC/10/CGRH/57).

## Results

### Resident Characteristics

**FIGURE 1:**
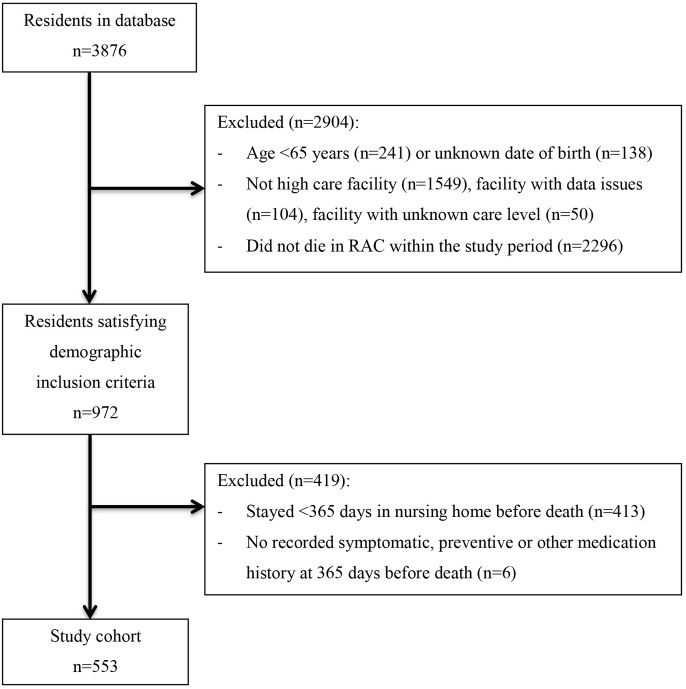
Flow chart of resident inclusion.

The cohort comprised of 553 residents out of the 3876 residents contained in the dataset, see **Figure 1**.

Residents were between 65 and 105 years of age and lived in 16 different facilities. The average facilities size was 35 (*SD*: 21) residents per facility (range: 5–71) (**Table 1**).

**Table 1:** Resident characteristics.

Characteristic	Residents
	(*n* = 553)
Age, mean years (*SD*)	88.0 (7.5)
Gender, % female (number)	68.1 (374)^∗^
Length of stay in RAC facility, mean weeks (*SD*)	187.9 (104.4)
Number of medications 365 days before death, mean (*SD*)	9.1 (4.5)
Number of medications at death, mean (*SD*)	8.7 (5.1)


^∗^*n = 549, gender was missing for four residents*.

### Number of Symptomatic, Preventive, and Other Medications in the Last Year of Life

**FIGURE 2:**
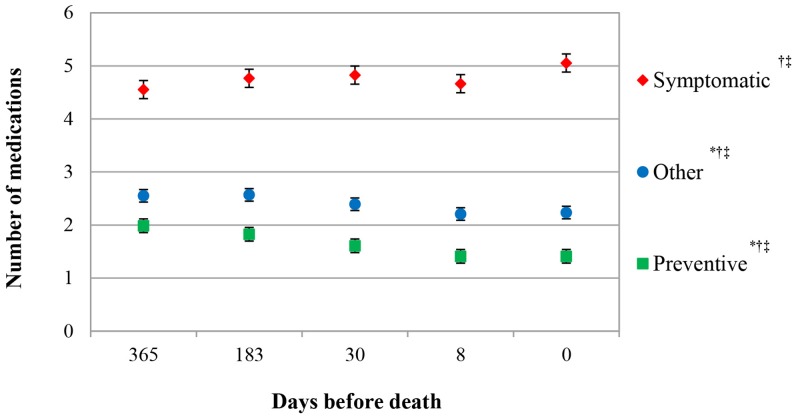
Number of symptomatic, preventive, and other medication in the last year of life. Estimated marginal means (EMMs), adjusted for number of bed days in facility^∗^ age^†^, and number of medication at 365 days before death^‡^.

The total number of medications per resident decreased from 9.1 [95% CI 8.9–9.3] medications 1 year prior to death to 8.5 [95% CI 8.5–8.9] medications at death (*P* = 0.002). Symptomatic medication use increased from 4.6 to 5.1 [95% CI 4.4–4.7 to 5.9–5.2, *P* = 0.000] medications, while preventive and other medication decreased, respectively 2.0 to 1.4 [95% CI 1.9–2.1 to 1.3–1.5, *P* = 0.000] and 2.6–2.2 [95% CI 2.4–2.7 to 2.1–2.4, *P* = 0.000], toward death (**Figure 2**).

### Type of Symptomatic, Preventive, and Other Medication Used in the Last Year of Life

Analgesics were the most frequently used type of medication over the last year of life. Analgesic use did not change significantly during the last year of life and was comparable at 1 year before death and at death (85.0 to 86.1% of patients, *P* = 0.610). A shift in the type of analgesics used was seen, shifting from paracetamol to opioids, respectively 83.4 to 77.9% (*P* = 0.005) and 18.1 to 44.5% (*P* = 0.000). Other significant changes in use of symptomatic medications toward death were only seen for diuretics (30.2 to 26.0%, *P* = 0.009) and medications for gastrointestinal disorders (17.2 to 22.8%, *P* = 0.000). In contrast, all preventive medications decreased significantly from 1 year before death until death. The highest decrease was found in mineral supplements (including calcium), agents acting on the renin-angiotensin-aldosterone-system (RAAS) and lipid modifying agents, those respectively decreased by 9.2% (*P* = 0.000), 8.9% (*P* = 0.000), and 8.1% (*P* = 0.000) (**Table 2**). However, at death about one third of all residents was using at least one antihypertensive medication (35.8%), one medication for osteoporosis (32.9%) or an antithrombotic medication (33.1%).

**Table 2:** Type of symptomatic, preventive, and other medication used by residents 1 year before death versus at death.

Symptomatic				Preventive				Other			
ATC code	Medication group	At death%	Δ %	ATC code	Medication group	At death %	Δ %	ATC code	Medication group	At death%	Δ %
N02	Analgesics	86.1	1.1	B01	Antithrombotics	33.1	-6.0^∗^	C01	Cardiac therapy	25.7	-2.7^∗^
A06	Laxatives	72.9	-0.4	A11	Vitamins	23.9	-5.6^∗^	N06	Psychoanaleptics	24.2	-6.0^∗^
N05	Psycholeptics	50.1	-0.4	C09	Agents acting on the RAAS system	21.3	-8.9^∗^	R03	Medication for obstructive airway disease	22.2	0.2
A02	Medication for acidic related disorders	38.3	-3.1	A12	Mineral supplements including calcium	17.9	-9.2^∗^	B03	Anti-anemic medication	15.9	-2.9
C03	Diuretics	26.0	-4.2^∗^	C07	Beta blockers	14.5	-2.7^∗^	A12	Mineral supplements (not including calcium)	14.6	-1.4
A03	Medication for gastrointestinal disorders	22.8	5.6^∗^	C10	Lipid modifying agents	9.9	-8.1^∗^	H03	Thyroid therapy	11.8	-1.1
N03	Antiepileptic medication	11.2	2.2	C08	Calcium channel blocking agents	7.1	-4.3^∗^	A10	Drugs used in diabetes	11.4	-3.4^∗^
H02	Corticosteroids for systemic use	8.3	-1.8	M05	Drugs for treatment of bone disease	5.6	-6.5^∗^				


*^∗^McNemar test (df = 552), *P* < 0.05. Δ: percentage of residents taking medication at death – percentage of residents taking medication 365 days before death. RAAS, renin-angiotensin-aldosterone-system.*

### Duration of Use of Symptomatic, Preventive, and Other Medications in the Last Year of Life

**FIGURE 3:**
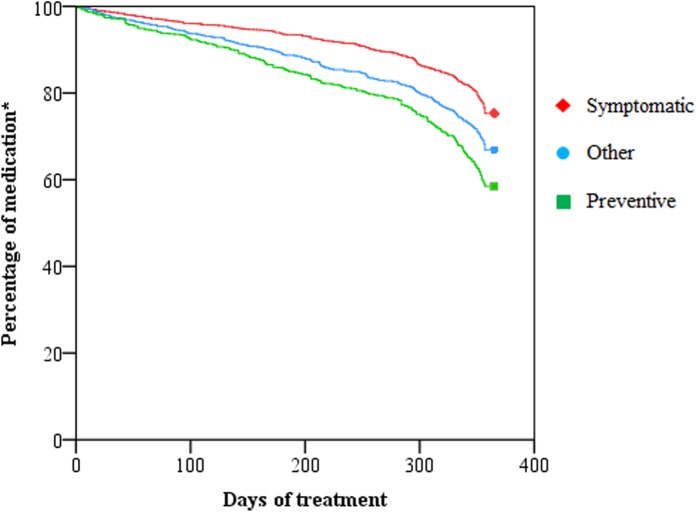
Duration of use of symptomatic, preventive and other medication in the last year of life. ^∗^We included all medications used 365 days before death.

Symptomatic, preventive, and other medications were used respectively for 336.3 [95% CI 331.8–340.8], 310.9 [95% CI 305.2–316.7] and 320.5 [95% CI 315.2–325.8] days in the last year of life. Preventive and other medications were ceased earlier than symptomatic medication, respectively 25.4 days earlier [EMM, 95% CI 31.0–19.7, *P* = 0.000] and 15.8 days earlier [EMM, 95% CI 20.9–10.7, *P* = 0.000] (**Figure 3**).

## Discussion

### Key Findings

Throughout the last year of life we saw little change in overall medication use. Medications commonly used for symptom control slightly increased, while a small decrease in medication for disease-prevention was seen. However at death, preventive medication such as antithrombotic agents, antihypertensive medications, and osteoporosis medications were still prescribed to one third of all residents.

### Changes in Medication Use at the End of Life

The characteristics of our cohort of residents are similar to other studies in this setting, so we believe our sample is representative for the nursing home population in Australia. The residents’ average duration of stay in the RAC facility was slightly higher than the national average (Australian Institute of Health and Welfare, 2017), which might be a consequence of selecting patients who stayed at least 1 year in the RAC.

We found an increase in symptomatic medication toward death, which was also seen in a small study looking at the last 3 months of life (Blass et al., 2008) and another study focusing at the last week of life (Jansen et al., 2014). The increase was very subtle, however, and mostly caused by an increase in gastrointestinal medications. Overall use of analgesics, which are supposed to be the most prominent medication group in palliative care (Australian Government Department of Health, 2015), did not change. But the shift from paracetamol to opioid use indicates some awareness in the changing needs of residents at the end of life by the GP.

Despite some deprescribing, the use of antithrombotics, antihypertensives, and osteoporosis medications was very high at the end of life, similar to other studies (Onder et al., 2013; Jansen et al., 2014; Heppenstall et al., 2016). An explanation for this high use could be the lack of consensus on what medications are considered solely preventive and therefore inappropriate at the end of life (Todd et al., 2017). We included antithrombotics, lipid-modifying agents, antihypertensives and osteoporosis medication, but other studies have also included iron, antibiotics, acid reducers and medications used in diabetes (Poudel et al., 2017). An exception to preventive medications, are lipid-modifying agents. These medications, especially statins, were unanimously classified as preventive medication and have been explored the most (Poudel et al., 2017). This attention to statins might have led to growing awareness of its inappropriateness at the end of life, resulting in early deprescribing by GPs. This could explain the lower use of statins compared to other preventive medication we found in our study.

### Strengths and Limitations

This study is unique in investigating changes in prescribing of symptomatic and preventive medication in the last year of life in a relatively large group of residents. We based the classification of medications on current guidelines. Some limitations need be taken into consideration when interpreting our results. Firstly, we were using medication supply data and therefore were not able to ascertain actual medication intake. However, the weekly medication supply ensured that the dataset remained relatively sensitive to change. Secondly, in line with other studies using dispensing data, we had no recorded indication for prescribed medication and therefore our medication classification was an approximation. We used the palliative care guidelines for classification of medication and rely on prescribing following the guidelines for correct classification. Thirdly, we were not able to cluster our data at the level of prescriber since each nursing home resident in Australia has his or her own prescriber. Fourthly, by investigating prescribing in the last year of life we had to exclude residents who stayed in the nursing home facility for a shorter time. Our results may not be generalizable to residents who died within a few months of nursing home admission.

## Conclusion and Implications for Further Research

The awareness of deprescribing inappropriate medication at the end of life is growing throughout the literature. Recent articles have been published guiding the process of deprescribing (Scott et al., 2015; Granas et al., 2017; Wouters et al., 2017) and shared decision making at the end of life (Jansen et al., 2016). But there still remains a lack of high quality evidence guiding deprescribing at the end of life (Tjia et al., 2013). For example aspirin has a number needed to treat of 120 patients over 6 years to prevent one cardiovascular event and a number needed to harm of 73 for a non-trivial bleedings, based on a study population with a mean age of 57 years (Seshasai et al., 2012). The figures are likely to be different in an older population. Furthermore, contradictory recommendations and variation in interpretations of guidelines leads to clinical uncertainty (Alhawassi et al., 2015). An example is the most recent discussion on blood pressure control in older patients (Williamson et al., 2016). Exploring the use of preventive and symptomatic medication use at the end of life is a first step to improve the quality of medication use for these patients.

## Author Contributions

LP was the initiator of the study, did the acquisition of the data and obtained ethical approval. HvdM, KT, and LP contributed to the conception and design of the study, the analysis and interpretation of the data. HvdM drafted the manuscript. All the authors revised the manuscript critically and approved the final manuscript.

## Conflict of Interest Statement

The authors declare that the research was conducted in the absence of any commercial or financial relationships that could be construed as a potential conflict of interest.

## Supplementary Material

APPENDIX S1 Classification of medications used by our cohort into symptomatic, preventive or other.
