# Supplementary material for: Changes in Prescribing Symptomatic and Preventive Medications in the Last Year of Life in Older Nursing Home Residents
Source: Front Pharmacol. 2018 Jan 23;8:990. doi: 10.3389/fphar.2017.00990 (PMC5787351; doi:10.3389/fphar.2017.00990)
Supplement: APPENDIX S1 — Classification of medications used by our cohort into symptomatic, preventive or other. [file Appendix.docx]

| **ATC code** | **Name** | **Category** |
| --- | --- | --- |
| A01AD11 | Various agents for local oral treatment | Other |
| A02AB01 | Aluminium Hydroxide | Other |
| A02AD01 | Ordinary salt combinations | Other |
| A02AF02 | Ordinary salt combinations and antiflatulents | Other |
| A02BA03 | Famotidine | Other |
| A02BX13 | Alginic acid | Other |
| A03AA04 | Mebeverine | Other |
| A03AX | Other drugs for functional gastrointestinal disorders | Other |
| A05BA03 | Silymarin | Other |
| A06AA | Softeners, emollients | Other |
| A06AC03 | Sterculia | Other |
| A07C | Electrolytes with carbohydrates | Other |
| A07EC01 | Sulfasalazine | Other |
| A07EC02 | Mesalazine | Other |
| A09A | Digestives, including enzymes | Other |
| A10AB | Fast-acting insulins | Other |
| A10AC | Intermediate-acting insulins | Other |
| A10AD | Intermediate- or long-acting combined with fast-acting insulins | Other |
| A10BA02 | Metformin | Other |
| A10BB01 | Glibenclamide | Other |
| A10BB07 | Glipizide | Other |
| A10BB09 | Gliclazide | Other |
| A10BG03 | Pioglitazone | Other |
| A11DA01 | Thiamine | Other |
| A11GB | Ascorbic acid, combinations | Other |
| A11JD | Other vitamin products, combinations | Other |
| A12BA | Potassium | Other |
| A12BA01 | Potassium chloride | Other |
| A12CA01 | Sodium chloride | Other |
| A12CB01 | Zinc sulfate | Other |
| A12CC | Magnesium | Other |
| A12CC05 | Magnesium aspartate | Other |
| B02BA01 | Phytomenadione | Other |
| B03A | Iron preparations | Other |
| B03BA01 | Cyanocobalamin | Other |
| B03BB | Folic acid | Other |
| B03XA02 | Darbepoetin alfa | Other |
| C01AA05 | Digoxin | Other |
| C01BC04 | Flecainide | Other |
| C01BD01 | Amiodarone | Other |
| C01CA24 | Epinephrine | Other |
| C01DA02 | Glyceryl Trinitrate | Other |
| C01DA08 | Isosorbide Dinitrate | Other |
| C01DA14 | Isosorbide Mononitrate | Other |
| C01DX16 | Nicorandil | Other |
| C01EB09 | Ubidecarenone | Other |
| G01AF02 | Clotrimazole | Other |
| G02CB03 | Cabergoline | Other |
| G03BA03 | Testosterone | Other |
| G03HA01 | Cyproterone | Other |
| G04BX | Sodium citrotartrate | Other |
| H03AA01 | Levothyroxine sodium | Other |
| H03BA02 | Propylthiouracil | Other |
| H03BB01 | Carbimazole | Other |
| H04AA01 | Glucagon | Other |
| J05AH02 | Oseltamivir | Other |
| L01AA02 | Chlorambucil | Other |
| L01BC02 | Fluorouracil | Other |
| L01BC06 | Capecitabine | Other |
| L01XX05 | Hydroxycarbamide | Other |
| L02AE02 | Leuprorelin | Other |
| L02AE03 | Goserelin | Other |
| L02BA01 | Tamoxifen | Other |
| L02BB02 | Nilutamide | Other |
| L02BG04 | Letrozole | Other |
| L02BG06 | Exemestane | Other |
| L03AB08 | Interferon beta-1b | Other |
| L04AX03 | Methotrexate | Other |
| M01AC01 | Piroxicam | Other |
| M01AC06 | Meloxicam | Other |
| M01AH01 | Celecoxib | Other |
| M01AX05 | Glucosamine | Other |
| M01AX25/M01AX05 | Chondroitin sulfate and Glucosamine | Other |
| M03BC01 | Orphenadrine citrate | Other |
| M04AA01 | Allopurinol | Other |
| M04AC01 | Colchicine | Other |
| N02AC04 | Dextropropoxyphene | Other |
| N02AC54 | Dextropropoxyphene, combincations excl. psycholeptics | Other |
| N02AX02 | Tramadol | Other |
| N02BA01 | Acetylsalicylic acid | Other |
| N03AA03 | Primidone | Other |
| N03AX09 | Lamotrigine | Other |
| N03AX14 | Levetiracetam | Other |
| N04AA01 | Trihexylphenidyl | Other |
| N04AA02 | Biperiden | Other |
| N04BA02 | Levodopa and decarboxylase inhibitor | Other |
| N04BA03 | Levodopa, decarboxylase inhibitor and COMT inhibitor | Other |
| N04BB01 | Amantadine | Other |
| N04BC02 | Pergolide | Other |
| N04BC05 | Pramipexole | Other |
| N04BC07 | Apomorphine | Other |
| N04BD01 | Selegine | Other |
| N04BX02 | Entacapone | Other |
| N05AB06 | Trifluoperazine | Other |
| N05AC01 | Pericyazine | Other |
| N05AC02 | Thioridazine | Other |
| N05AF01 | Flupenthixol | Other |
| N05AH04 | Quetiapine | Other |
| N05AN | Lithium | Other |
| N05AX12 | Aripiprazole | Other |
| N05BA08 | Bromazepam | Other |
| N05CF01 | Zopiclone | Other |
| N05CF02 | Zolpidem | Other |
| N06AA02 | Imipramine | Other |
| N06AA16 | Dosuleptin | Other |
| N06AB03 | Fluoxetine | Other |
| N06AB04 | Citalopram | Other |
| N06AB06 | Sertraline | Other |
| N06AB08 | Fluvoxamine | Other |
| N06AB10 | Escitalopram | Other |
| N06AF03 | Phenelzine | Other |
| N06AG02 | Moclobemide | Other |
| N06AX03 | Mianserin | Other |
| N06AX11 | Mirtazapine | Other |
| N06AX18 | Reboxetine | Other |
| N06AX23 | Desvenlafaxine | Other |
| N06BA07 | Modafinil | Other |
| N06DA02 | Donepezil | Other |
| N06DA03 | Rivastigmine | Other |
| N06DA04 | Galantamine | Other |
| N06DX01 | Memantine | Other |
| N07BA01 | Nicotine | Other |
| N07CA01 | Betahistine | Other |
| P01BA02 | Hydroxychloroquine | Other |
| P01BC01 | Quinine | Other |
| P02CF01 | Ivermectin | Other |
| P03AC04 | Permethrin | Other |
| R03BA07 | Mometasone | Other |
| R02AA03 | Dichlorobenzyl alcohol | Other |
| R02AD02 | Lidocaine | Other |
| R03AC02 | Salbutamol | Other |
| R03AC03 | Terbutaline | Other |
| R03AK06 | Fluticasone and Salmeterol | Other |
| R03AK07 | Formoterol and Budesonide | Other |
| R03BA01 | Beclomethasone | Other |
| R03BA02 | Budesonide | Other |
| R03BA05 | Fluticasone | Other |
| R03BB01 | Ipratropium bromide | Other |
| R03BB04 | Tiotropium bromide | Other |
| R03DA04 | Theophylline | Other |
| R05CA12 | Hederae helicis folium | Other |
| R05CB02 | Bromhexine | Other |
| R05DA04 | Codeine | Other |
| R05DA08 | Pholcodine | Other |
| R05DA09 | Dextromethorphan | Other |
| R05DA12 | Acetyldihydrocodeine | Other |
| R06AA02 | Diphenhydramine | Other |
| R06AB02 | Dexchlorpheniramine | Other |
| R06AE07 | Cetirizine | Other |
| R06AX02 | Cyproheptadine | Other |
| R06AX13 | Loratadine | Other |
| R06AX26 | Fexofenadine | Other |
| V03AB33 | Hydroxycobolamin | Other |
| V03AE01 | Polystyrene Sulfonate | Other |
| V03AG | Sodium celulose phosphate | Other |
| A11CC | Vitamine D and vitamine D analogues | Preventive |
| A12A | Calcium | Preventive |
| B01 | Antithrombotic agents | Preventive |
| C02 | Antihypertensives | Preventive |
| C03 | Diuretics (except for hydrochlorothiazide, frusemide, spironolactone) | Preventive |
| C07 | Betablocking agents | Preventive |
| C08 | Calcium channel blockers (except for nifedipine and diltiazem) | Preventive |
| C09 | Agents acting on the renin angiotensin system | Preventive |
| C10A | Lipid modifying agents (plain) | Preventive |
| C10B | Lipid modifying agents (combinations) | Preventive |
| G03C | Estrogen | Preventive |
| G03F | Progesteron | Preventive |
| G03XC01 | Raloxifene hydrochloride | Preventive |
| G04CA03 | Terazosin hydrochloride | Preventive |
| H05AA02 | Teriparatide | Preventive |
| H05BA | Calcitonin | Preventive |
| H05BX01 | Cinacalcet | Preventive |
| M05BA | Bisphosphonates (except for clodronic acid, pamidromic acid, ibedronic acid, zoledronic acid) | Preventive |
| M05BB | Bisphosphonates combinations | Preventive |
| M05BX03 | Strontium ranelate | Preventive |
| M05BX04 | Denosumab (calcium & bone metabolism medicines) | Preventive |
| A01AD02 | Benzydamine | Symptomatic |
| A02BA02 | Ranitidine | Symptomatic |
| A02BC01 | Omeprazole | Symptomatic |
| A02BC02 | Pantoprazole | Symptomatic |
| A02BC03 | Lansoprazole | Symptomatic |
| A02BC04 | Rabeprazole | Symptomatic |
| A02BC05 | Esomeprazole | Symptomatic |
| A02BX02 | Sucralfate | Symptomatic |
| A03AB02 | Glycooyrronium bromide | Symptomatic |
| A03AB05 | Propantheline | Symptomatic |
| A03BA01 | Atropine sulfate | Symptomatic |
| A03FA01 | Metoclopramide | Symptomatic |
| A03FA02 | Cisapride | Symptomatic |
| A03FA03 | Domperidone | Symptomatic |
| A04AA01 | Ondansetron | Symptomatic |
| A04AA02 | Granisetron | Symptomatic |
| A04AA03 | Tropisetron | Symptomatic |
| A04AA04 | Dolasetron | Symptomatic |
| A04AD01 | Hyoscine hydrobromide | Symptomatic |
| A04AD10 | Dronabinol | Symptomatic |
| A04AD11 | Nabilone | Symptomatic |
| A04AD12 | Aprepitant | Symptomatic |
| A06AA01 | Liquid paraffin | Symptomatic |
| A06AA02 | Docusate | Symptomatic |
| A06AB02 | Bisacodyl | Symptomatic |
| A06AB06 | Senna glycosides | Symptomatic |
| A06AB08 | Sodium picosulphate | Symptomatic |
| A06AB56 | Senna glycosides combinations | Symptomatic |
| A06AC01 | Ispaghula (psylla seeds) | Symptomatic |
| A06AC53 | Stericula combinations | Symptomatic |
| A06AD11 | Lactulose | Symptomatic |
| A06AD15 | Macrogol | Symptomatic |
| A06AD17 | Sodium phosphate | Symptomatic |
| A06AD18 | Sorbitol | Symptomatic |
| A06AG11 | Sorbitol Lauryl Sulfoacetate and combinations | Symptomatic |
| A06AH01 | Methylnaltrexone | Symptomatic |
| A06AH04 | Naloxone | Symptomatic |
| A06AX01 | Glycerol | Symptomatic |
| A07DA03 | Loperamide | Symptomatic |
| A09AA02 | Pancrelipase | Symptomatic |
| A10AE04 | Long acting insulin | Symptomatic |
| B02AA02 | Tranexamic acid | Symptomatic |
| B05B | I.V. solutions | Symptomatic |
| C01BB02 | Mexiletine | Symptomatic |
| C03AA03 | Hydrochlorothiazide | Symptomatic |
| C03CA01 | Furosemide | Symptomatic |
| C03DA01 | Spironolactone | Symptomatic |
| C08CA05 | Nifedipine | Symptomatic |
| C08DB01 | Diltiazem | Symptomatic |
| G04BD04 | Oxybutynin | Symptomatic |
| G04BD08 | Solifenacin succinate | Symptomatic |
| G04CA02 | Tamsulosin | Symptomatic |
| G04CB01 | Finasteride | Symptomatic |
| H01CB02 | Octreotide | Symptomatic |
| H01CB03 | Lanreotide | Symptomatic |
| H02AA02 | Fludrocortisone | Symptomatic |
| H02AB02 | Dexamethasone | Symptomatic |
| H02AB04 | Methylprednisolone | Symptomatic |
| H02AB06 | Prednisolone | Symptomatic |
| H02AB07 | Prednisone | Symptomatic |
| H02AB09 | Hydrocortisone | Symptomatic |
| H02AB10 | Cortisone acetate | Symptomatic |
| J02AC01 | Fluconazole oral | Symptomatic |
| J02AC02 | Itraconazole oral | Symptomatic |
| J05AB01 | Aciclovir (i.v.) | Symptomatic |
| J05AB09 | Famciclovir | Symptomatic |
| J05AB11 | Valaciclovir | Symptomatic |
| M01AB01 | Indomethacin | Symptomatic |
| M01AB05 | Diclofenac | Symptomatic |
| M01AB55 | Diclofenac combinations | Symptomatic |
| M01AE01 | Ibuprofen | Symptomatic |
| M01AE02 | Naproxen | Symptomatic |
| M03BX01 | Baclofen | Symptomatic |
| M03CA01 | Dantrolene | Symptomatic |
| M05BA02 | Clodronic acid | Symptomatic |
| M05BA03 | Pamidronic acid | Symptomatic |
| M05BA06 | Ibedronic acid | Symptomatic |
| M05BA08 | Zoledronic acid | Symptomatic |
| Mouthwash | Bioactive enzymes mouthwash | Symptomatic |
| N01AH03 | Sufentanil | Symptomatic |
| N01AX03 | Ketamine | Symptomatic |
| N01BB02 | Lignocaine | Symptomatic |
| N02AA01 | Morphine hydrochloride | Symptomatic |
| N02AA03 | Hydromorphone | Symptomatic |
| N02AA05 | Oxycodone | Symptomatic |
| N02AB03 | Fentanyl | Symptomatic |
| N02AE01 | Buprenorphine | Symptomatic |
| N02BE01 | Paracetamol | Symptomatic |
| N02BE51 | Codeine | Symptomatic |
| N03AB02 | Phenytoin | Symptomatic |
| N03AE01 | Clonazepam | Symptomatic |
| N03AF01 | Carbamazepine | Symptomatic |
| N03AG01 | Sodium Valproate | Symptomatic |
| N03AX12 | Gabapentin | Symptomatic |
| N03AX16 | Pregabalin | Symptomatic |
| N04AC01 | Benzatropine | Symptomatic |
| N05AA01 | Chlorpromazine | Symptomatic |
| N05AA02 | Levomepromazine | Symptomatic |
| N05AB04 | Prochlorperazine | Symptomatic |
| N05AD01 | Haloperidol | Symptomatic |
| N05AH03 | Olanzapine | Symptomatic |
| N05AX08 | Risperidone | Symptomatic |
| N05BA01 | Diazepam | Symptomatic |
| N05BA04 | Oxazepam | Symptomatic |
| N05BA06 | Lorazepam | Symptomatic |
| N05BA12 | Alprazolam | Symptomatic |
| N05CD02 | Nitrazepam | Symptomatic |
| N05CD07 | Temazepam | Symptomatic |
| N05CD08 | Midazolam | Symptomatic |
| N06AA09 | Amitriptyline | Symptomatic |
| N06AA10 | Nortriptyline | Symptomatic |
| N06AA12 | Doxepin | Symptomatic |
| N06AB05 | Paroxetine | Symptomatic |
| N06AX16 | Venlafaxine | Symptomatic |
| N06AX21 | Duloxetine | Symptomatic |
| N06BA02 | Dexamfetamine | Symptomatic |
| N06BA04 | Methylphenidate | Symptomatic |
| N07BC02 | Methdadone | Symptomatic |
| R06AD01 | Alimemazine | Symptomatic |
| R06AD02 | Promethazine | Symptomatic |
| R06AE03 | Cyclizine | Symptomatic |
